# Has vitamin D had its day? An audit of vitamin D, prolactin and HBA1C monitoring over one year in an in-patient secure service

**DOI:** 10.1192/bjo.2021.868

**Published:** 2021-06-18

**Authors:** Oleg Lujanschi, Toral Thomas

**Affiliations:** 1 Northside House, Medium Secure Unit; 2 Northside House MSU

## Abstract

**Aims:**

To ensure that service users in in-patient secure services have prolactin, vitamin D and HbA1c monitoring as per current best practice guidance.

**Background:**

Service users prescribed antipsychotic medication are at risk of developing raised prolactin levels and metabolic syndrome. In both sexes, long-standing hyperprolactinaemia can lead to low bone mineral density with an increased risk of developing osteoporosis.

In recent years there has been increasing controversy on the increase in Vitamin D monitoring despite the poor evidence for complications from vitamin D deficiency in adults. Not undertaking this test in the absence of symptoms will potentially reduce anxiety for service users could save £17 per test and £50 for a 12-week course of Vitamin D supplementation. Local and national guidance indicate Vitamin D monitoring should only be done in symptomatic people.

**Method:**

Fifty-five service users in the five in-patient wards had their electronic records and pathology results reviewed over a one-year period. All service users were expected to have a minimum of an annual HbA1c and prolactin level but to only have vitamin D monitoring if symptomatic for deficiency.

**Result:**

Although 100% of service users in MSU were tested, vitamin D testing was consistently undertaken without documented clinical evidence of deficiency. The ranges across all units were: prolactin (72- 1384mU/L), HbA1c (30–90 mmol/mol) and vitamin D (15–124 nmol/L). Local reference ranges are prolactin (53- 360mU/L), HbA1c (<48 mmol/mol) and Vitamin D (50–120 nmol/L).

Prolactin levels were highest on the male medium secure wards.

The other two units had significantly less testing with prolactin and HbA1c levels being the least measured (18% of service users on male LSU and 23% on the female ward respectively). Vitamin D testing on these two wards were 38% on the female ward and 18% on the male ward for both tests.

**Conclusion:**

Northside House has a dedicated physical health team and this is likely to explain its 100% score. However, vitamin D testing was being undertaking automatically rather than based on symptoms.

The recommendation is to add prolactin and HbA1c to the physical screens done before CPA meetings for all service users prescribed an antipsychotic but to stop Vitamin D testing in the absence of clinical symptoms of vitamin D deficiency.

